# Susceptibility of Domestic Goat (*Capra aegagrus hircus*) to Experimental Infection with Severe Acute Respiratory Syndrome Coronavirus 2 (SARS-CoV-2) B.1.351/Beta Variant

**DOI:** 10.3390/v14092002

**Published:** 2022-09-09

**Authors:** Leira Fernández-Bastit, Núria Roca, Miguel Romero-Durana, Jordi Rodon, Guillermo Cantero, Óscar García, Carlos López, Mònica Pérez, Rosa López, Jorge Carrillo, Nuria Izquierdo-Useros, Julià Blanco, Bonaventura Clotet, Joan Pujols, Júlia Vergara-Alert, Joaquim Segalés, Cristina Lorca-Oró

**Affiliations:** 1 Unitat Mixta d’Investigació IRTA-UAB en Sanitat Animal, Centre de Recerca en Sanitat Animal (CReSA), Campus de la Universitat Autònoma de Barcelona (UAB), 08193 Bellaterra, Spain; 2 IRTA, Programa de Sanitat Animal, Centre de Recerca en Sanitat Animal (CReSA), Campus de la Universitat Autònoma de Barcelona (UAB), 08193 Bellaterra, Spain; 3 Life Sciences Department, Joint BSC-CRG-IRB Research Program in Computational Biology, Barcelona Supercomputing Center, 08034 Barcelona, Spain; 4 IrsiCaixa AIDS Research Institute, 08916 Badalona, Spain; 5 Germans Trias i Pujol Research Institute (IGTP), Can Ruti Campus, 08916 Badalona, Spain; 6 Infectious Disease Networking Biomedical Research Center (CIBERINFEC), Carlos III Health Institute, 28029 Madrid, Spain; 7 Infectious Diseases and Immunity, Faculty of Medicine, University of Vic-Central University of Catalonia (UVic-UCC), 08500 Barcelona, Spain; 8 Lluita Contra la SIDA Foundation, Hospital Universitari Germans Trias i Pujol, 08916 Badalona, Spain; 9 Departament de Sanitat i Anatomia Animals, Facultat de Veterinària, Universitat Autònoma de Barcelona, 08193 Cerdanyola del Vallès, Spain

**Keywords:** severe acute respiratory syndrome coronavirus 2 (SARS-CoV-2), Beta variant, goat, ruminant, susceptibility, experimental infection

## Abstract

A wide range of animal species are susceptible to the severe acute respiratory syndrome coronavirus 2 (SARS-CoV-2) infection. Natural and/or experimental infections have been reported in pet, zoo, farmed and wild animals. Interestingly, some SARS-CoV-2 variants, such as B.1.1.7/Alpha, B.1.351/Beta, and B.1.1.529/Omicron, were demonstrated to infect some animal species not susceptible to classical viral variants. The present study aimed to elucidate if goats (*Capra aegagrus hircus*) are susceptible to the B.1.351/Beta variant. First, an in silico approach was used to predict the affinity between the receptor-binding domain of the spike protein of SARS-CoV-2 B.1.351/Beta variant and angiotensin-converting enzyme 2 from goats. Moreover, we performed an experimental inoculation with this variant in domestic goat and showed evidence of infection. SARS-CoV-2 was detected in nasal swabs and tissues by RT-qPCR and/or immunohistochemistry, and seroneutralisation was confirmed via ELISA and live virus neutralisation assays. However, the viral amount and tissue distribution suggest a low susceptibility of goats to the B.1.351/Beta variant. Therefore, although monitoring livestock is advisable, it is unlikely that goats play a role as SARS-CoV-2 reservoir species, and they are not useful surrogates to study SARS-CoV-2 infection in farmed animals.

## 1. Introduction

The causative agent of the coronavirus infectious disease 2019 (COVID-19) pandemic, severe acute respiratory syndrome coronavirus 2 (SARS-CoV-2), is mainly transmitted via respiratory droplets or aerosols from human to human [1]. Additionally, SARS-CoV-2 can infect several animal species that may act as intermediate hosts and play a role in the transmission and maintenance of the virus. Considering that the origin of this pandemic is apparently zoonotic [2,3,4], it is important to determine the risk of infection in susceptible species, as well as the risk of reverse zoonosis [5,6]. Moreover, to better understand the epidemiology of this zoonotic pathogen under the “One Health” approach, it is crucial to identify the susceptibility of animal species and to investigate if they could act as potential reservoirs and/or intermediate hosts, especially those that are in close contact with human populations as domestic livestock.

In an increasingly connected world, the emergence of multiple variants since the start of the COVID-19 pandemic has urged the implementation of rapid measures to control the spreading of the virus. Several variants of concern (VOCs) have been identified with different infectivity or transmissibility properties in humans and animal species. As of 31st August 2022, more than 599 million cases in humans and 700 outbreaks or infection events in animals, involving 23 species in 36 countries, have been reported [7,8]. Natural SARS-CoV-2 infections were reported in zoo animals (i.e., felids, but also gorillas, otters, and white-tailed deer, among others), farmed animals (mainly minks), and pets (i.e., cats, dogs, ferrets and hamsters) [9,10,11,12]. The susceptibility of non-human primates, hamsters, ferrets, cats, deer, rabbits, raccoon dogs, fruit bats, and skunks has also been demonstrated by experimental infections [13,14,15,16,17,18,19]. Importantly, wild-type mice are not susceptible to early pandemic variants of SARS-CoV-2 under experimental conditions, but susceptible to certain variants such as B.1.1.7/Alpha, BA.1.1/Omicron, and especially B.1.351/Beta [20,21,22,23]. This latter variant was first reported in South Africa in late 2020 and showed nine mutations in the spike (S) protein compared to the ancestral variant [24], allowing it to partially escape from previously acquired neutralising antibodies (nAbs) against SARS-CoV-2 and to increase its transmissibility. Indeed, the described novel viral variants acquired mutations along the genome, but most importantly in the receptor-binding domain (RBD) of the SARS-CoV-2 S protein which is responsible for the recognition of the angiotensin-converting enzyme 2 (ACE2) host receptor [25,26]. The affinity between the RBD and the ACE2 receptor as well as the distribution in different tissues of the ACE2 in animal species is a putative indicator of host susceptibility to infection [27]. These differences between variants raise concerns regarding how the risk of new mutations in SARS-CoV-2 could increase susceptibility in novel species, with the danger of becoming zoonotic reservoirs. 

The interactions between livestock animals and wildlife increase cross-transmission of infectious diseases which can reduce food safety and also result in economic impact [28]. Among ruminants, white-tailed deer has been found to be the most susceptible species to ancestral and Alpha SARS-CoV-2 variants, showing high seroprevalence in free-ranging animals in the USA [29]. Moreover, subclinical infection has been demonstrated following experimental challenge, as well as viral transmissibility between in-contact white-tailed deer and vertically from doe to foetus [15,30]. Regarding other ruminant species, sheep, cattle, and goat have shown low susceptibility to SARS-CoV-2 ancestral variant [31] or the combination of ancestral plus Alpha/B.1.1.7 variants [32]. The domestic goat (*Capra aegagrus hircus*) is a small ruminant species raised worldwide in a broad range of production systems for its meat, milk, hide, and hair [33]. Since goats are in constant contact with humans and other SARS-CoV-2 susceptible animal species, it is of high interest to investigate their susceptibility to this viral infection. As mentioned previously, previous studies reported limited susceptibility of livestock (cattle, sheep, and goats) to SARS-CoV-2 USA-WA1/2020 reference isolate [31,32] under experimental conditions; however, the emergence of new SARS-CoV-2 variants raises the possibility of host range expansion [20,34]. Since the potential reservoirs and intermediate hosts of SARS-CoV-2 are still being investigated and little information is available regarding the susceptibility of livestock to different variants, the aim of the present study was to evaluate the susceptibility of the domestic goat to the B.1.351/Beta variant of SARS-CoV-2, one of the most important VOCs that already showed expanded species tropism [21,23,34].

## 2. Materials and Methods

### 2.1. In Silico Study

We ran MODELLER v10.1 [35] to generate ten different homology models of goat ACE2 in complex with the spike RBD of the SARS-CoV-2 ancestral variant. The crystallographic structure of human ACE2 in complex with the spike RBD of the ancestral variant (PDB ID 6M0J) was used as a template. Then, using the MODELLER models as input, we used FoldX v5 [36] to predict the changes in binding affinity induced by the mutations of the variants. We considered the following mutations located at the RBD: K417N, E484K, N501Y for B.1.351/Beta, L452R, T478K for B.1.617.2/Delta, K417T, E484K, N501Y for P.1/Gamma, and G339D, S371L, S373P, S375F, K417N, N440K, G446S, S477N, T478K, E484A, Q493R, G496S, Q498R, N501Y, Y505H for B.1.1.529/Omicron (BA.1) variants. We ran FoldX modules repairPDB followed by BuildModel. We fixed all FoldX parameters to their default values except parameter vdwDesign, which was set to zero. As a positive control, we performed the same steps using the human ACE2 sequence to predict the changes in binding affinity caused by the mutations of the SARS-CoV-2 VOC in humans.

### 2.2. Cells and Virus

Vero E6 cells (ATCC^®^ repository, CRL-1586™) and SARS-CoV-2 B.1.351/Beta variant isolate (passage 3; hCoV-19/Spain/CT-IrsiCaixaR008CC8B3/2021; GISAID ID EPI_ISL_3164134) were prepared as previously reported [37] in Dulbecco’s modified Eagle Medium, DMEM (Lonza, Basel, Switzerland) supplemented with 5% foetal calf serum (FCS; EuroClone, Milan, Italy), 100 U/mL penicillin, 100 μg/mL streptomycin, and 2 mM glutamine (all from Gibco Life Technologies, Madrid, Spain).

The infectious titre of the SARS-CoV-2 stock was calculated by determining the dilution that caused cytopathic effect (CPE) in 50% of the inoculated Vero E6 cells (50% tissue culture infectious dose endpoint, TCID_50_).

### 2.3. Experimental Study Design

This experimental study was approved by the Institutional Animal Welfare Committee of the Institute of Agrifood Research and Technology (CEEA-IRTA) and by the Ethical Commission of Animal Experimentation of the Autonomous Government of Catalonia (CEA-OH/11586/3) and conducted by certified staff. Experiments involving SARS-CoV-2 were performed at the Biosafety Level-3 (BSL-3) facilities of the Biocontainment Unit of IRTA-CReSA (Barcelona, Spain).

**Figure 1:**
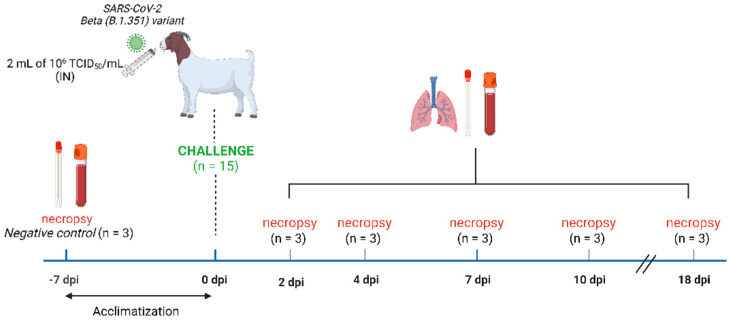
Schematic representation of the study design. Three out of eighteen goats were necropsied on the day of arrival and served as non-inoculated controls. Nasal and rectal swabs, blood, and tissue samples were collected. After acclimatisation, the remaining fifteen goats were intranasally inoculated with the SARS-CoV-2 B.1.351/Beta variant (2 mL of 1 × 10^6^ TCID_50_/animal). At 2, 4, 7,10, and 18 days post-infection, three goats were necropsied each day and nasal and rectal swabs, blood, and tissues were collected. Clinical signs and rectal temperature were recorded daily.

A total of 18 goats of 2–3 months of age were acquired from a Spanish commercial farm (La botiga d’Ullastrell S.L, Barcelona, Spain) and included in the study. Three animals were used as non-inoculated controls and were necropsied on the day of animal arrival. The remaining 15 animals were allocated inside an experimental box of the animal BSL-3 facilities, and after 7 days of acclimation, they were intranasally (IN) inoculated with 2 mL of 10^6^ TCID_50_/mL of SARS-CoV-2 B.1.351/Beta variant (1 mL in each nostril) using a diffusor device (LMA™ MAD^®^ Nasal, Teleflex LLC). At 2, 4, 7, 10 and 18 days post-inoculation (dpi), three goats/day were euthanised and complete necropsies were performed (Figure 1). At necropsy, nasal and rectal swabs (NS/RS), blood, and the following tissues were collected: nasal turbinate (NT; caudal and cranial portions), olfactory bulb, parotid gland, trachea, pharynx, tonsil, lung (apical, medial, and caudal portions), lymph nodes (LN; cervical, mesenteric, and mediastinal ones), kidney, spleen, jejunum, colon, and heart. Clinical signs and rectal temperatures were recorded daily during the whole study.

### 2.4. Virus Detection and Isolation

Tissues, NS, and RS were collected during necropsy and were transferred into cryotubes containing 500 µL DMEM supplemented with 100 U/mL penicillin, 100 μg/mL streptomycin, and 2 mM glutamine, vortexed and stored at −75 °C until use. Blood was centrifuged (1800× *g*, 10 min at 4 °C) and sera were collected and kept at −20 °C until further use. Detection of SARS-CoV-2 RNA was performed via genomic and subgenomic reverse transcription-quantitative PCR (RT-qPCR) in all collected samples, as previously described [38,39]. Briefly, viral RNA was extracted using the Indimag Pathogen Kit (Indical Biosciences, Leipzig, Germany) on a Biosprint 96 workstation (Qiagen, Hilden, Germany) in accordance with the manufacturers’ instructions. Then, RT-qPCR was carried out using AgPath-IDTM One-Step RT-PCR Reagents (Applied Biosystems, Life Technologies), and amplification was performed using a 7500 Fast Real-Time PCR System (Applied Biosystems, Life Technologies, Waltham, MA, USA). Samples with a Ct value < 40 were considered positive for SARS-CoV-2 RNA.

RT-qPCR positive samples with Ct < 30 were evaluated for the presence of infectious virus by titration in Vero E6 cells, as previously reported [37]. Briefly, ten-fold dilutions from each sample were transferred to Vero E6 monolayers and incubated at 37 °C and 5% CO_2_. Plates were monitored under a light microscope and wells were assessed for the presence of CPE for 6 days. The amount of infectious virus was calculated by determining the TCID_50_ using the Reed and Muench method [40].

### 2.5. Neutralising Antibodies

Blood samples of the 18 experimental goats were centrifuged at 1800× *g* for 10 min at 4 °C, and then the obtained sera were inactivated at 56 °C for 1 h. Neutralising antibodies targeting SARS-CoV-2 RBD from all samples were measured with the GenScript cPass^TM^ SARS-CoV-2 Neutralization Antibody Detection Kit (Genscript, the Netherlands), following the manufacturer’s protocol. The percentage of inhibition of each sample was calculated by the following formula: % Inhibition = (1 − (OD_450_ sample/OD_450_ negative control)) × 100. Samples and controls were included in duplicate (SD ≤ 10%). Inhibition of ≥ 30% was considered a positive neutralisation.

In addition, live virus neutralisation assay was performed as previously described [37]. Pre-inactivated serum samples were initially diluted 1:20 and then 2-fold serially diluted in supplemented DMEM, mixed 1:1 with the SARS-CoV-2 isolate (EPI_ISL_3164134), and incubated for 1 h at 37 °C. Then, each dilution mixture (in duplicates) was transferred to Vero E6 monolayers containing 100 TCID_50_ of SARS-CoV-2 per well and cultured for 3 days at 37 °C and 5% CO_2_. Then, CPE was measured using the CellTiter-Glo luminescent cell viability assay (Promega, Madison, WI, USA) in accordance with the manufacturer’s protocol. Luminescence was measured as luminescence units in a Fluoroskan Ascent FL luminometer (ThermoFisher Scientific, Waltham, MA, USA). Serum virus neutralisation titre 50 (SNT_50_) was defined as the reciprocal value of the sample dilution that showed 50% of the SARS-CoV-2-induced CPE in Vero E6 cells.

### 2.6. Histopathology

Upper (NT) respiratory tract and tonsil were harvested and fixed by immersion in 10% buffered formalin solution. Fixed tissues were embedded in paraffin, and cut sections (3 µm thick) were stained with haematoxylin and eosin (H/E) for histopathology studies using an optical microscope.

A previously described immunohistochemistry (IHC) technique to detect SARS-CoV-2 NP antigen [41] using a Rabbit monoclonal antibody (40143-R019, SinoBiological, Beijing, China) at dilution 1:10,000 was applied on NT and tonsil. The amount of viral antigen in tissue samples was semi-quantitatively scored: lack of antigen detection, low, moderate, and high amount following a previously published scoring system [37].

### 2.7. Statistical Analyses

Dose–response curves of neutralisation assay in serum samples were adjusted to a non-linear fit regression model calculated with a normalised logistic curve with variable slope. For data normalisation, uninfected cells and untreated virus-infected cells were used as negative and positive control of infection (% Neutralisation = (RLUmax − RLUexperimental)/(RLUmax − RLUmin) × 100), respectively. All SNT_50_ values were determined as the concentration of sera blocking 50% of the SARS-CoV-2-induced CPE in Vero E6 cells and were expressed as reciprocal dilution. All statistical analyses were performed with GraphPad Prism 8.4.3 (GraphPad Software, Inc., San Diego, CA, USA).

## 3. Results

### 3.1. In Silico Predictions of Binding Affinity Changes in B.1.351/Beta, B.1.617.2/Delta, P.1/Gamma, and B.1.1.529/Omicron Variants

**Figure 2:**
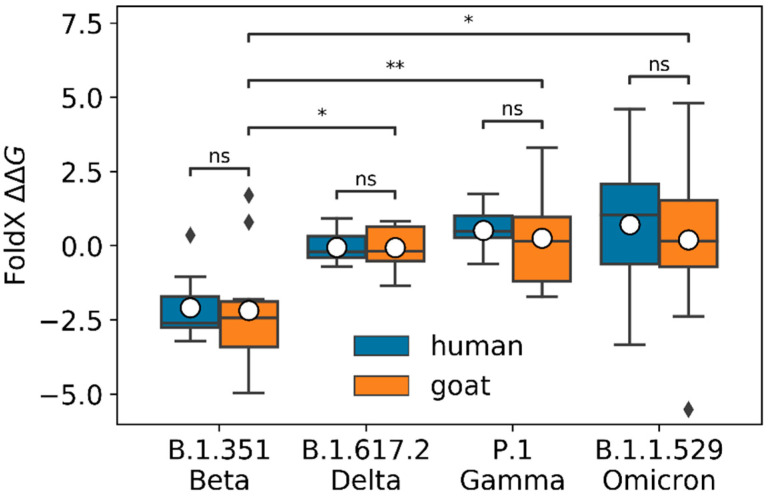
FoldX predicted ∆∆G for B.1.351/Beta, B.1.617.2/Delta, P.1/Gamma, and B.1.1.529/Omicron variants for human and goat ACE2 (blue and orange boxes, respectively). For goat, the computed ∆∆G was significantly lower for the Beta variant than for the Delta, Gamma, and Omicron variants, with Mann–Whitney–Wilcoxon test *p*-values of $1.06\times 10^{-2}$, $3.64\times 10^{-3}$, and $3.20\times 10^{-2}$, respectively. The average ∆∆G predicted from the 10 Modeller models is plotted as a white dot for each variant. We found no significant differences between the predicted ∆∆G values for human and goat of the different variants (Mann–Whitney–Wilcoxon test *p*-values of $4.85\;\times 10^{-1},\;5.15\;\times \;10^{-1},\;7.64\;\times \;10^{-1},\;6.61\times \;10^{-1}$ for Delta, Beta, Gamma, and Omicron, respectively). In this boxplot representation, the lower and upper ends of each box represent the first (Q1) and third (Q3) quartiles of the ∆∆G predicted values, respectively. The horizontal line, inside each box, represents the median, or second quartile (Q2), and the mean is plotted as a white dot for each variant. The box “whiskers” extend to values that are 1.5 times the size of the interquartile range (IQR = Q3 − Q1). Values that fall outside this range are displayed independently as black diamonds. Mann-Whitney-Wilcoxon test *p*-values are annotated according to the following criteria: ns (0.05 < *p*-value ≤ 1), * (0.02 < *p*-value ≤ 0.05), ** (0.001 < *p*-value ≤ 0.02).

We ran FoldX to predict the changes in binding affinity ($\Delta \Delta G={\Delta G}_{\mathrm{variant}}-{\Delta G}_{\mathrm{ancestral}}$) for the spike RBD in complex with goat ACE2, induced by the mutations of the variants we studied (see Figure 2). As a control, we also computed the binding affinity changes for the spike RBD in complex with human ACE2. Results for goat ACE2 indicated that Beta ∆∆G was significantly lower (higher binding affinity changes) than Delta, Gamma, and Omicron ∆∆Gs (Mann–Whitney–Wilcoxon test *p*-values equal to 1.06 × 10^−2^, 3.64 × 10^−3^, and 3.20 × 10^−2^, respectively). We found a similar trend for human ACE2 (Mann–Whitney–Wilcoxon test *p*-values equal to 6.58 × 10^−4^, 2.91 × 10^−4^, and 1.06 × 10^−2^ for Delta, Gamma, and Omicron, respectively). Positive/negative ∆∆Gs values indicated if the binding affinity was lower/higher for the evaluated variant in comparison with the ancestral spike RBD in complex with goat ACE2. The average ∆∆Gs for the Beta variant was −2.18, with a 95% confidence interval (CI) of (−3.67, −0.69), for the Delta variant −0.06, with a 95% CI of (−0.58, 0.46), for the Gamma variant 0.25 with a 95% CI of (0.97, 1.48), and for the Omicron variant 0.20, with a 95% CI of (−1.86, 2.26). We did not find significant differences between the predicted ∆∆Gs for goat and human for any of the variants (Mann–Whitney–Wilcoxon test *p*-values of $4.85\;\times \;10^{-1}$, $5.15\;\times \;10^{-1}$, $7.64\;\times 10^{-1}$, and $6.61\;\times \;10^{-1}$ for Delta, Beta, Gamma, and Omicron, respectively).

### 3.2. Clinical Signs and Pathological Findings

No clinical signs or increase in rectal temperature from baseline were observed after challenge in any of the goats along the study. Moreover, no gross or microscopic lesions attributable to SARS-CoV-2 infection were recorded in any of the studied animals.

### 3.3. Virus Detection and Replicative Viral Isolation in Goat Samples

**Figure 3:**
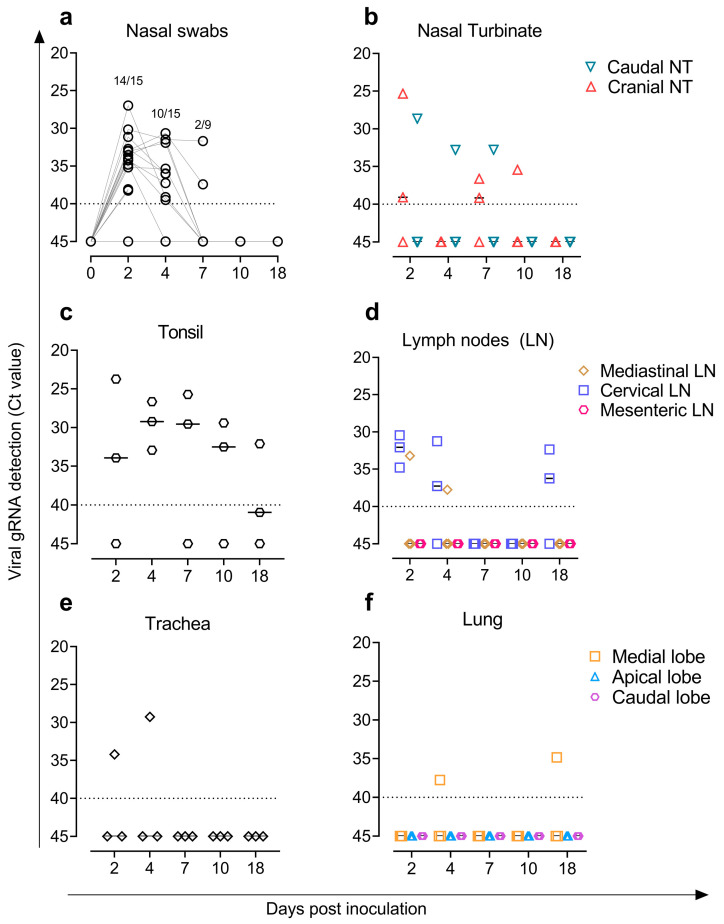
Detection of SARS-CoV-2 genomic RNA (gRNA) by RT-qPCR. SARS-CoV-2 loads in (**a**) nasal swabs; (**b**) cranial and caudal nasal turbinate; (**c**) tonsil; (**d**) mediastinal, cervical, and mesenteric lymph nodes; (**e**) trachea; and (**f**) lung. Horizontal bars indicate median viral loads. Dotted lines reflect the limit of detection (Ct = 40).

Fourteen out of the fifteen goats tested positive for SARS-CoV-2 by genomic RT-qPCR in NS at 2 dpi and ten out of twelve at 4 dpi with low viral loads (Ct ≥ 27). Only two out of the nine remaining goats were positive with low viral loads (Ct > 30) at 7 dpi (Figure 3a). Moreover, low levels of viral RNA (Ct > 23) were observed in caudal and cranial NT (Figure 3b), lymphoid tissues (tonsil and cervical and/or mediastinal LN) (Figure 3c,d), and respiratory tract (trachea and lung) (Figure 3e,f). SARS-CoV-2 RNA from tonsil and LN samples were detected until 18 dpi in goats (Figure 3c,d). Regarding subgenomic RNA, tonsil samples were positive at 2, 4, and 7 dpi (Ct > 23) as well as cranial and caudal NT at 2 dpi (Ct > 25). Blood and all other samples (RS, olfactory bulb, parotid gland, trachea, spleen, kidney, jejunum, and heart) tested negative (Ct ≥ 40) via genomic and subgenomic RT-qPCR.

Only samples with a Ct < 30 by RT-qPCR were tested for virus titration on cell culture (Table S1). We isolated infectious virus on Vero E6 cells at 2 dpi from one tonsil (3.1 TCID_50_/mL) and one cranial NT (1.9 TCID_50_/mL) from two different goats. SARS-CoV-2 could not be isolated from all other tested samples.

### 3.4. Detection of SARS-CoV-2 Nucleoprotein in Tissues by Immunohistochemistry

**Figure 4:**
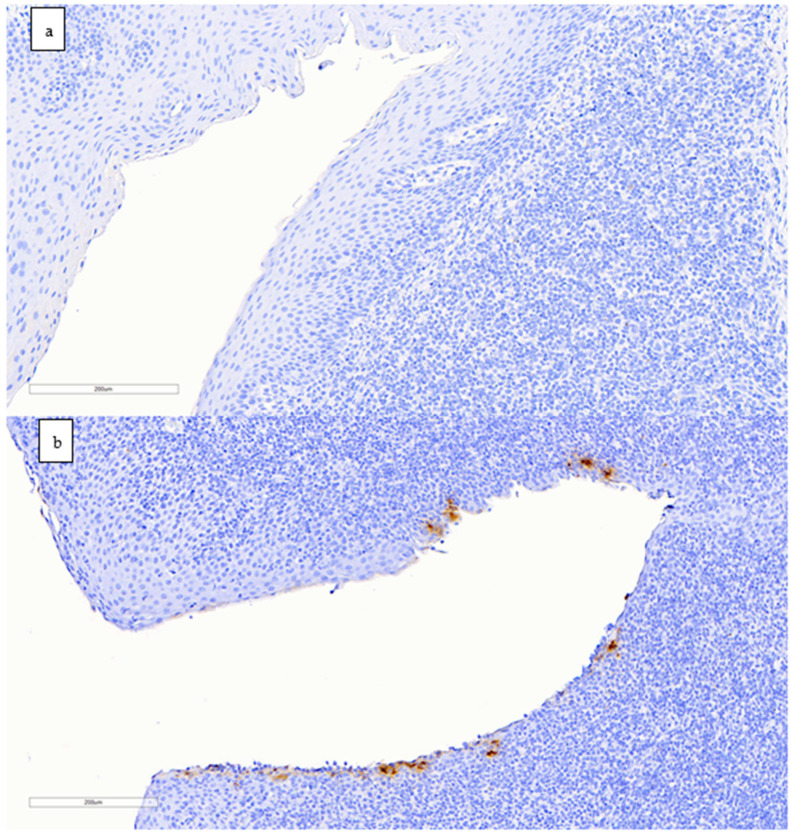
Immunohistochemistry staining to detect the nucleocapsid protein of SARS-CoV-2 in goat tonsils (scale bar: 200 µm). (**a**) Negative control animal with no antigen labelling. (**b**) Positive result in the tonsil of a goat euthanised at 2 dpi; immunolabelling is seen as brownish staining in dendritic-like cells around a tonsillar crypt.

Tonsils from inoculated goats collected at 2, 4, and 7 dpi (one goat per day) were positive for IHC staining (Figure 4); labelling was mainly found in dendritic-like cells mostly located around tonsillar crypts. The amount of viral antigen was low in all animals. All other samples were negative.

### 3.5. Inoculated Goats Developed Humoral Responses against SARS-CoV-2

**Figure 5:**
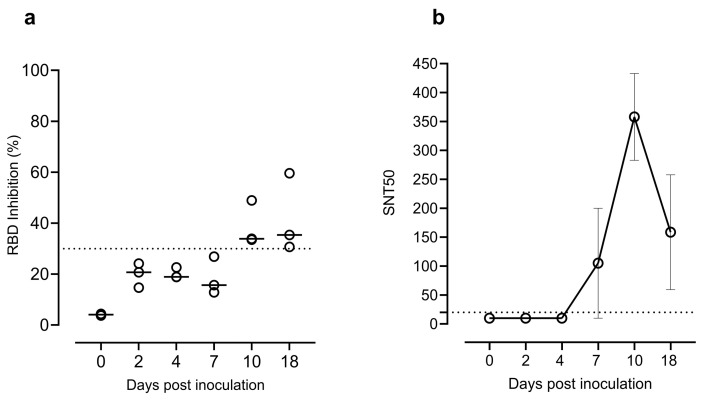
(**a**) Neutralising antibodies detected by the SARS-CoV-2 RBD Inhibition ELISA (Positive ≥ 30% RBD inhibition). (**b**) Neutralisation titres in sera samples from 0, 2, 4, 7, 10, and 18 dpi determined by the live virus neutralisation assay. Data reported as values of reciprocal dilution of SNT_50_ (mean ± SEM). The horizontal dotted lines indicate the cut-off value of the assay. Abbreviations: RBD, receptor-binding domain; SNT_50_, serum virus neutralisation titre (reciprocal dilution) that showed 50% protection of virus growth.

Neutralising antibodies targeting the RBD of SARS-CoV-2 were detected in goats at 10 and 18 dpi by the RBD Inhibition ELISA assay (Figure 5a). Seroneutralisation capacity against SARS-CoV-2 B.1.351/Beta variant was observed at 7, 10, and 18 dpi by the live virus neutralisation assay (Figure 5b).

## 4. Discussion

Goat is an important livestock animal that has already been suggested to be susceptible to SARS-CoV-2 by previous studies, both in vitro and in vivo [31,42]. Similarity between goat and human ACE2 receptors has been demonstrated via comparative genomic analysis as well as binding affinity between goat ACE2 and SARS-CoV-2 S protein [42]. Other previous in vitro studies also reported that goat ACE2 supports cell entry of SARS-CoV-2 [43,44], and its replication has been demonstrated in DF1 cells expressing both goat ACE2 and transmembrane serine protease 2 (TMPRSS2) [43]. Since the susceptibility of animal species could change after the appearance of SARS-CoV-2 variants and our in silico study predicted potential affinity between the B.1.351/Beta variant and ACE2 goat receptor, the aim of the present study was to investigate the susceptibility of goat to this variant of SARS-CoV-2 by means of an in vivo experimental inoculation.

Previously, Bosco-Lauth et al. [31] described SARS-CoV-2 RT-qPCR positive results in two out of three inoculated goats at 3 days after experimental challenge with the ancestral variant. However, none of the animals shed detectable infectious virus, nor did other ruminants such as sheep and cattle. The aforementioned study together with other previous studies reported that goat, sheep, or cattle developed limited subclinical infection after SARS-CoV-2 inoculation [20,32]. In sheep, Gaudreault et al. [32] isolated SARS-CoV-2 from trachea samples at 4 dpi, while RNA was detected in respiratory and lymphoid tissues at 4, 8 and 21 dpi. Meanwhile, Ulrich et al. [45] did not perform virus isolation from RT-qPCR positive samples obtained from SARS-CoV-2 inoculated cattle. Although our study performed in goats was carried out with a higher sample size than the mentioned experimental infections in livestock ruminants, comparable results regarding overall susceptibility in goats were observed. Low amounts of viral genome and antigen in tissues were found in the inoculated goats. Although the detected nucleic acid may be considered remnants of the inoculum, the detection of subgenomic RNA in NT and tonsil samples at 2 dpi suggests recent virus replication, at least in those tissues. In addition, the virus isolation from these tissues supports the presence of infectious virus in this species after challenge with the B.1.351/Beta variant. On the other hand, results from IHC exhibited presence of nucleocapsid protein of SARS-CoV-2 in the tonsil of a single goat at each of the time points (2, 4, and 7 dpi) in cells with morphology compatible with dendritic cells (DCs). It is likely that this immunolabelling represents viral internalisation and accumulation in these cells, since at least human DCs have been shown to trap SARS-CoV-2 even though not showing a productive infection [46,47]. Further studies would be needed to understand whether viral replication or DC internalisation occur in tonsils in this or other potentially susceptible species.

Differences in viral loads observed in our study compared to those of previous experimental infections in goats, as well as in the other ruminant species, may be due to the higher affinity predicted for B.1.351/Beta variant in goats than for ancestral variants [31,32]. This may confirm that the emergence of new variants raises concerns regarding the risk of new mutations in SARS-CoV-2 that could increase the range of susceptibility of zoonotic reservoirs.

In our study, the dose of challenge may be higher than the one that goats could be exposed to under natural conditions. Thus, it is unknown whether inoculation with a lower dose of virus would have shown evidence of infection or not. In a parallel study, we tested 208 goats that had been in contact with COVID-19 positive farmers, yet none of the goats developed humoral immune responses against SARS-CoV-2 (unpublished work) supporting that they were not exposed to the virus albeit the circumstances.

Overall, our results suggest that domestic goat has low susceptibility to SARS-CoV-2 B.1.351/Beta variant infection with low amounts of viral genome and antigen in tissues and evidence of seroconversion from 7 dpi onwards. Moreover, challenged goats did not show clinical signs or gross and microscopic lesions consistent with SARS-CoV-2 infection. Thus, the domestic goat seems to be a poorly competent host for SARS-CoV-2 (at least for the Beta variant) and probably has a negligible role in virus transmission, in agreement with studies carried out in other ruminant livestock species [31,32,46]. However, it is important to continue monitoring potential susceptible wild and domestic species to minimize the risk of transmission at the human-animal interface. Moreover, the circulating variants such as Omicron and the emergence of new ones raise concerns regarding the risk of new mutations in SARS-CoV-2 that could expand host range susceptibility and generate new zoonotic reservoirs. In fact, a recent study with 27 ACE2 orthologues including goat suggests a broader species receptor binding of Omicron compared to other variants such as Delta [48]. Thus, the infectivity of new viral variants in different animal species should be continuously monitored as improved viral replication cannot be ruled out in species currently defined as poorly susceptible.

## Data Availability

The SARS-CoV-2 B.1.351/Beta variant isolate information used as challenge in this study has been deposited in GISAID under accession number EPI_ISL_3164134 (passage 3; hCoV-19/Spain/CT-IrsiCaixaR008CC8B3/2021).

## Supplementary Materials

The following are available online at https://www.mdpi.com/article/10.3390/v14092002/s1, Supplementary Table S1. Results obtained by viral titration from those samples with a Ct < 30 as measured by genomic RT-qPCR.

## References

[1] Jayaweera M., Perera H., Gunawardana B., Manatunge J. (2020). Transmission of COVID-19 Virus by Droplets and Aerosols: A Critical Review on the Unresolved Dichotomy. Environ. Res..

[2] Temmam S., Vongphayloth K., Salazar E.B., Munier S., Bonomi M., Regnault B., Douangboubpha B., Karami Y., Chrétien D., Sanamxay D. (2022). Bat Coronaviruses Related to SARS-CoV-2 and Infectious for Human Cells. Nature.

[3] Zhou P., Yang X.L., Wang X.G., Hu B., Zhang L., Zhang W., Si H.R., Zhu Y., Li B., Huang C.L. (2020). A Pneumonia Outbreak Associated with a New Coronavirus of Probable Bat Origin. Nature.

[4] Worobey M., Levy J.I., Serrano L.M., Crits-Christoph A., Pekar J.E., Goldstein S.A., Rasmussen A.L., Kraemer M.U.G., Newman C., Koopmans M.P.G. (2022). The Huanan Seafood Wholesale Market in Wuhan Was the Early Epicenter of the COVID-19 Pandemic. Science.

[5] Sharun K., Dhama K., Pawde A.M., Gortázar C., Tiwari R., Katterine Bonilla-Aldana D., Rodriguez-Morales A.J., De La Fuente J., Michalak I., Attia Y.A. (2021). SARS-CoV-2 in Animals: Potential for Unknown Reservoir Hosts and Public Health Implications. Vet. Q..

[6] Dróżdż M., Krzyżek P., Dudek B., Makuch S., Janczura A., Paluch E. (2021). Current State of Knowledge about Role of Pets in Zoonotic Transmission of SARS-CoV-2. Viruses.

[7] World Health Organization Tracking SARS-CoV-2 Variants. https://www.who.int/activities/tracking-SARS-CoV-2-variants.

[8] World Organisation for Animal Health (WOAH) Events in Animals. https://www.woah.org/en/what-we-offer/emergency-and-resilience/covid-19/#ui-id-3.

[9] Fernández-Bellon H., Rodon J., Fernández-Bastit L., Almagro V., Padilla-Solé P., Lorca-Oró C., Valle R., Roca N., Grazioli S., Trogu T. (2021). Monitoring Natural SARS-CoV-2 Infection in Lions (*Panthera leo*) at the Barcelona Zoo: Viral Dynamics and Host Responses. Viruses.

[10] Goraichuk I.V., Arefiev V., Stegniy B.T., Gerilovych A.P. (2021). Zoonotic and Reverse Zoonotic Transmissibility of SARS-CoV-2. Virus Res..

[11] McAloose D., Laverack M., Wang L., Killian M.L., Caserta L.C., Yuan F., Mitchell P.K., Queen K., Mauldin M.R., Cronk B.D. (2020). From People to Panthera: Natural Sars-Cov-2 Infection in Tigers and Lions at the Bronx Zoo. mBio.

[12] Munnink B.B.O., Sikkema R.S., Nieuwenhuijse D.F., Molenaar R.J., Munger E., Molenkamp R., Van Der Spek A., Tolsma P., Rietveld A., Brouwer M. (2021). Transmission of SARS-CoV-2 on Mink Farms between Humans and Mink and Back to Humans. Science.

[13] Munster V.J., Feldmann F., Williamson B.N., Van Doremalen N., Pérez-Pérez L., Schulz J., Meade-White K., Okumura A., Callison J., Brumbaugh B. (2020). Respiratory Disease in Rhesus Macaques Inoculated with SARS-CoV-2. Nature.

[14] Shi J., Wen Z., Zhong G., Yang H., Wang C., Huang B., Liu R., He X., Shuai L., Sun Z. (2020). Susceptibility of Ferrets, Cats, Dogs, and Other Domesticated Animals to SARS-Coronavirus 2. Science.

[15] Palmer M.V., Martins M., Falkenberg S., Buckley A., Caserta L.C., Mitchell P.K., Cassmann E.D., Rollins A., Zylich N.C., Renshaw R.W. (2021). Susceptibility of White-Tailed Deer (*Odocoileus virginianus*) to SARS-CoV-2. J. Virol..

[16] Schlottau K., Rissmann M., Graaf A., Schön J., Sehl J., Wylezich C., Höper D., Mettenleiter T.C., Balkema-Buschmann A., Harder T. (2020). SARS-CoV-2 in Fruit Bats, Ferrets, Pigs, and Chickens: An Experimental Transmission Study. Lancet Microbe.

[17] Bosco-Lauth A.M., Root J.J., Porter S.M., Walker A.E., Guilbert L., Hawvermale D., Pepper A., Maison R.M., Hartwig A.E., Gordy P. (2021). Peridomestic Mammal Susceptibility to Severe Acute Respiratory Syndrome Coronavirus 2 Infection. Emerg. Infect. Dis..

[18] Freuling C.M., Breithaupt A., Müller T., Sehl J., Bakema-Buschmann A., Rissmann M., Klein A., Wylezich C., Höper D., Wernike K. (2020). Susceptibility of Raccoon Dogs for SARS-CoV-2. Emerg. Infect. Dis..

[19] Mykytyn A.Z., Lamers M.M., Okba N.M.A., Breugem T.I., Schipper D., van den Doel P.B., van Run P., van Amerongen G., de Waal L., Koopmans M.P.G. (2021). Susceptibility of Rabbits to SARS-CoV-2. Emerg. Microbes Infect..

[20] Tarrés-Freixas F., Trinité B., Pons-Grífols A., Romero-Durana M., Riveira-Muñoz E., Ávila-Nieto C., Pérez M., Garcia-Vidal E., Perez-Zsolt D., Muñoz-Basagoiti J. (2022). Heterogeneous Infectivity and Pathogenesis of SARS-CoV-2 Variants Beta, Delta and Omicron in Transgenic K18-HACE2 and Wildtype Mice. Front. Microbiol..

[21] Pan T., Chen R., He X., Yuan Y., Deng X., Li R., Yan H., Yan S., Liu J., Zhang Y. (2021). Infection of Wild-Type Mice by SARS-CoV-2 B.1.351 Variant Indicates a Possible Novel Cross-Species Transmission Route. Signal. Transduct. Target. Ther..

[22] Zhang Y.N., Zhang Z.R., Zhang H.Q., Li N., Zhang Q.Y., Li X.D., Deng C.L., Deng F., Shen S., Zhu B. (2022). Different Pathogenesis of SARS-CoV-2 Omicron Variant in Wild-Type Laboratory Mice and Hamsters. Signal. Transduct. Target. Ther..

[23] Shuai H., Chan J.F.W., Yuen T.T.T., Yoon C., Hu J.C., Wen L., Hu B., Yang D., Wang Y., Hou Y. (2021). Emerging SARS-CoV-2 Variants Expand Species Tropism to Murines. EBioMedicine.

[24] Tegally H., Wilkinson E., Giovanetti M., Iranzadeh A., Fonseca V., Giandhari J., Doolabh D., Pillay S., San E.J., Msomi N. (2021). Detection of a SARS-CoV-2 Variant of Concern in South Africa. Nature.

[25] Hoffmann M., Kleine-Weber H., Schroeder S., Krüger N., Herrler T., Erichsen S., Schiergens T.S., Herrler G., Wu N.H., Nitsche A. (2020). SARS-CoV-2 Cell Entry Depends on ACE2 and TMPRSS2 and Is Blocked by a Clinically Proven Protease Inhibitor. Cell.

[26] Wan Y., Shang J., Graham R., Baric R.S., Li F. (2020). Receptor Recognition by the Novel Coronavirus from Wuhan: An Analysis Based on Decade-Long Structural Studies of SARS Coronavirus. J. Virol..

[27] Zhai X., Sun J., Yan Z., Zhang J., Zhao J., Zhao Z., Gao Q., He W.-T., Veit M., Su S. (2020). Comparison of Severe Acute Respiratory Syndrome Coronavirus 2 Spike Protein Binding to ACE2 Receptors from Human, Pets, Farm Animals, and Putative Intermediate Hosts. J. Virol..

[28] Jori F., Hernandez-Jover M., Magouras I., Dürr S., Brookes V.J. (2021). Wildlife-Livestock Interactions in Animal Production Systems: What Are the Biosecurity and Health Implications?. Anim. Front..

[29] Hale V.L., Dennis P.M., Mcbride D.S., Nolting J.M., Madden C., Huey D., Ehrlich M., Grieser J., Winston J., Lombardi D. (2022). SARS-CoV-2 Infection in Free-Ranging White-Tailed Deer. Nature.

[30] Cool K., Gaudreault N.N., Morozov I., Trujillo J.D., Meekins D.A., McDowell C., Carossino M., Bold D., Kwon T., Balaraman V. (2022). Infection and Transmission of Ancestral SARS-CoV-2 and Its Alpha Variant in Pregnant White-Tailed Deer. bioRxiv.

[31] Bosco-Lauth A.M., Walker A., Guilbert L., Porter S., Hartwig A., Mcvicker E., Bielefeldt-Ohmann H., Bowen R.A. (2021). Susceptibility of Livestock to SARS-CoV-2 Infection. Emerg. Mcirobes Infect..

[32] Gaudreault N.N., Cool K., Trujillo J.D., Morozov I., Meekins D.A., McDowell C., Bold D., Carossino M., Balaraman V., Mitzel D. (2022). Susceptibility of Sheep to Experimental Co-Infection with the Ancestral Lineage of SARS-CoV-2 and Its Alpha Variant. Emerg. Microbes Infect..

[33] Gilbert M., Nicolas G., Cinardi G., Van Boeckel T.P., Vanwambeke S.O., Wint G.R.W., Robinson T.P. (2018). Global Distribution Data for Cattle, Buffaloes, Horses, Sheep, Goats, Pigs, Chickens and Ducks in 2010. Nat. Sci. Data.

[34] Montagutelli X., Prot M., Levillayer L., Salazar E.B., Jouvion G., Conquet L., Donati F., Albert M., Gambaro F., Behillil S. (2021). The B1.351 and P.1 Variants Extend SARS-CoV-2 Host Range to Mice. bioRxiv.

[35] Šali A., Blundell T.L. (1993). Comparative Protein Modelling by Satisfaction of Spatial Restraints. J. Mol. Biol..

[36] Schymkowitz J., Borg J., Stricher F., Nys R., Rousseau F., Serrano L. (2005). The FoldX Web Server: An Online Force Field. Nucleic Acids Res..

[37] Brustolin M., Rodon J., Rodríguez de la Concepción M.L., Ávila-Nieto C., Cantero G., Pérez M., Te N., Noguera-Julián M., Guallar V., Valencia A. (2021). Protection against Reinfection with D614- or G614-SARS-CoV-2 Isolates in Golden Syrian Hamster. Emerg. Microbes Infect..

[38] Wölfel R., Corman V.M., Guggemos W., Seilmaier M., Zange S., Müller M.A., Niemeyer D., Jones T.C., Vollmar P., Rothe C. (2020). Virological Assessment of Hospitalized Patients with COVID-2019. Nature.

[39] Corman V.M., Landt O., Kaiser M., Molenkamp R., Meijer A., Chu D.K.W., Bleicker T., Brünink S., Schneider J., Schmidt M.L. (2020). Detection of 2019 Novel Coronavirus (2019-NCoV) by Real-Time RT-PCR. Eurosurveillance.

[40] Reed L.J., Muench H. (1938). A Simple Method of Estimating Fifty per Cent Endpoints. Am. J. Epidemiol..

[41] Rockx B., Kuiken T., Herfst S., Bestebroer T., Lamers M.M., Munnink B.B.O., De Meulder D., Van Amerongen G., Van Den Brand J., Okba N.M.A. (2020). Comparative Pathogenesis of COVID-19, MERS, and SARS in a Nonhuman Primate Model. Science.

[42] Damas J., Hughes G.M., Keough K.C., Painter C.A., Persky N.S., Corbo M., Hiller M., Koepfli K.-P., Pfenning A.R., Zhao H. (2020). Broad Host Range of SARS-CoV-2 Predicted by Comparative and Structural Analysis of ACE2 in Vertebrates. Proc. Natl. Acad. Sci. USA.

[43] Kapczynski D.R., Sweeney R., Spackman E., Pantin-Jackwood M., Suarez D.L. (2022). Development of an in Vitro Model for Animal Species Susceptibility to SARS-CoV-2 Replication Based on Expression of ACE2 and TMPRSS2 in Avian Cells. Virology.

[44] Zhang H.L., Li Y.M., Sun J., Zhang Y.Y., Wang T.Y., Sun M.X., Wang M.H., Yang Y.L., Hu X.L., Tang Y.D. (2021). Evaluating Angiotensin-Converting Enzyme 2-Mediated SARS-CoV-2 Entry across Species. J. Biol. Chem..

[45] Ulrich L., Wernike K., Hoffmann D., Mettenleiter T.C., Beer M. (2020). Experimental Infection of Cattle with SARS-CoV-2. Emerg. Infect. Dis..

[46] Niles M.A., Gogesch P., Kronhart S., Ortega Iannazzo S., Kochs G., Waibler Z., Anzaghe M. (2021). Macrophages and Dendritic Cells Are Not the Major Source of Pro-Inflammatory Cytokines Upon SARS-CoV-2 Infection. Front. Immunol..

[47] Perez-Zsolt D., Muñoz-Basagoiti J., Rodon J., Elosua-Bayes M., Raïch-Regué D., Risco C., Sachse M., Pino M., Gumber S., Paiardini M. (2021). SARS-CoV-2 Interaction with Siglec-1 Mediates Trans-Infection by Dendritic Cells. Joaquim Segalés.

[48] Li L., Han P., Huang B., Xie Y., Li W., Zhang D., Han P., Xu Z., Bai B., Zhou J. (2022). Cell Discovery Broader-Species Receptor Binding and Structural Bases of Omicron SARS-CoV-2 to Both Mouse and Palm-Civet ACE2s. Cell Discov..

