# Supplementary material for: Susceptibility of Domestic Goat (Capra aegagrus hircus) to Experimental Infection with Severe Acute Respiratory Syndrome Coronavirus 2 (SARS-CoV-2) B.1.351/Beta Variant
Source: Viruses. 2022 Sep 9;14(9):2002. doi: 10.3390/v14092002 (PMC9503527; doi:10.3390/v14092002)
Supplement: Supplementary file 1 [file viruses-14-02002-s001.zip › viruses-1877967-supplementary.pdf]

Goat	DPI	Tissue	RT-qPCR, genomic RNA (Ct <30)	Virus isolation (log10 $TCID_{50}$/mL)
1	2	Tonsil	23.73	3.1
2	2	NT caudal	28.66	<1.8
2	2	NT cranial	25.32	1.9
4	4	Tonsil	26.64	<1.8
6	4	Tonsil	29.22	<1.8
8	7	Tonsil	25.71	<1.8
9	7	Tonsil	29.55	<1.8
10	10	Tonsil	29.42	<1.8
11	2	Nasal swab	26.96	<1.8

Supplementary Table S1. Results obtained by viral titration from those samples with a Ct < 30 by genomic RT-qPCR.
